# Detecting statistical interactions in immune receptor data: a comparative study

**DOI:** 10.1080/02664763.2025.2533483

**Published:** 2025-07-27

**Authors:** Thomas Minotto, Ingrid Hobæk Haff, Enrico Riccardi, Geir K. Sandve

**Affiliations:** a Department of Mathematics, University of Oslo, Oslo, Norway; b Department of Informatics, University of Oslo, Oslo, Norway

**Keywords:** Antibody-antigen binding, interaction detection, logic regression, logistic regression, machine learning, random forests, 62J07

## Abstract

Statistical interactions are part of numerous data generating processes and several methods have been developed to detect them. We here study immune receptors binding to antigens, where advanced machine learning techniques have proved useful for binding prediction, suggesting significant intra amino acid chain interactions. We reviewed detection methods based on logistic lasso, logic regression, random forests and neural networks. We compared detection performance in simulated immune data, and how it is affected by the order of interactions, their strength related to the main effects, their frequency of occurrence and the size of the data. Interactions were implanted as motifs of amino acids that determined the binding status of sequences through a logistic regression model. Results show that pairwise interactions were retrieved from just 1000 sequences in the dataset, and optimal detection happened for an implantation rate of around 20 percent. For higher-order interactions, the best performance was obtained by logic regression and random forest based methods. The running time for the neural network-based method was several orders of magnitude lower, followed by the lasso-based methods. We applied the methods on an experimental dataset and identified several pairwise interactions as well as a three-way interaction, enhancing the accuracy of prediction models.

## 1. Introduction

Detecting statistical interactions is a significant aspect of data analysis as it helps establishing correct interpretation of the data generating process. Covariates may be correlated and interact together to create the response variable, in which case failing to take into account interactions might lead to incomplete models and nonoptimal accuracy in prediction tasks. For instance, in cancer susceptibility studies, important progress has been enabled by including interactions between groups of alleles in addition to the main effects, as discussed in [11]. In health studies, the effect of mixtures of pollutants was shown to be more important than what the effect of single pollutant could suggest, for example in [14]. For the particular case of categorical data, several authors have proposed approaches to detect statistical interactions. Ritchie *et al.* [28] have introduced Multifactor Dimensionality Reduction, inspired by combinatorial techniques, that enables to identify interactions in gene data. Nunkesser *et al.* [22] develop GPAS (Genetic Programming for Association Studies), an algorithm based on genetics to detect interactions between genes in SNPs (Single Nucleotide Polymorphisms) data. A review paper [3] investigates the ability of several methods to detect interactions in exposome data. Interactions between continuous variables have also been widely explored, but are not the focus of this study.

While other interaction detection methods have been developed that can handle more diverse type of categorical or binary data, and applied to biological datasets with success [3], an interesting and unexplored case where statistical interactions are of crucial importance is the study of how adaptive immune receptors bind to antigens. Antibodies and antigens are chains of amino acids that can interact with each other and, eventually, bind. The process involves several contributions, including inter chain interactions [1] and long-range interactions inside antibody chains [8]. The complexity of the binding process requires advanced machine learning techniques to be predicted. Convolutional Neural Networks (CNN), Long Short Term Memory networks (LSTM), Support Vector Machines, random forests and regularized logistic regression models have successfully predicted binding status in both simulated and experimental data (see, e.g. [17,21,27,30,34,35]). Yet, most of these methods do not allow a direct interpretation of the underlying processes. To deconstruct the respective importance of individual amino acids and groups of amino acids along the chain, we propose here to determine the cases where statistical interactions can be detected and used to model immune receptor binding to antigens.

Our study is based on data generated in silico using a probability distribution model of V(D)J gene recombination, a biological mechanism by which the vertebrate immune system creates various immune receptors to fight against pathogens [37]. We created several data sets where interactions between amino acids determine whether the antibody sequences should be binders or non-binders in the model, and compared the performance of several interaction detection methods. The methods reviewed are two based on logistic-lasso [6,19], three based on logic regression [16,18,31], one based on random forest [4] and one based on neural networks [38]. We selected methods that work with binary covariates and response, can handle several hundred covariates, and have open access code for the implementation. This research has shown what kind of statistical interactions can be detected in immune receptor data and with which methods. To our knowledge, there have not been previous attempts at detecting statistical interactions in immune receptor data, nor at focusing specifically on interaction detection instead of prediction of the response. Further, the detection methods that we used have not been compared with each other before, neither applied to this field.

In Section 2, we present each of these interaction detection method. In Section 3, we introduce different models to construct datasets with main effects and interactions, and present the simulation study. Section 4 focuses on the results of the study and the comparison of interaction detection methods. Section 5 gives an illustration on experimental data.

## 2. Methods

We aim to study immune receptor binding, which can be formulated as predicting P(Y=1 | X=x), where X is an amino acid sequence, and *Y* is a response variable indicating whether the sequence binds to an antigen (*Y* = 1) or not ( Y=0). For simplicity, amino acid sequences are assumed to be of fixed length *L*, such that each $X_{j},j=1,\ldots ,L$ is originally a categorical covariate with 20 categories, one per possible amino acid. Further, we assume that the data generating mechanism is a generalised additive model [15], where the log-odds depend on a linear function *F* of covariates and products of covariates

g(P(Y=1 | X=x))=F(x),

where g:(0,1)→R is the logit function $g(x)=\log\frac{x}{1-x}$.

As stated by [36], the function F(x), where $x=(x_{1},x_{2},\ldots ,x_{p})$, shows no pairwise interaction between variables $x_{i_{1}}$ and $x_{i_{2}}$ if it can be expressed as the sum of two functions, $f_{\setminus i_{1}}$ and $f_{\setminus i_{2}}$, that do not depend on $x_{i_{1}}$ and $x_{i_{2}}$, respectively, i.e.

$$F(x)=f_{\setminus i_{1}}(x_{1},\ldots ,x_{i_{1}-1},x_{i_{1}+1},\ldots ,x_{p})+f_{\setminus i_{2}}(x_{1},\ldots ,x_{i_{2}-1},x_{i_{2}+1},\ldots ,x_{p}).$$

For example, $F(x_{1},x_{2},x_{3})=\sin(x_{1}+x_{2})+x_{1}x_{3}$ has interactions between $x_{1}$ and $x_{2}$ and also between $x_{1}$ and $x_{3}$, but no interaction between $x_{2}$ and $x_{3}$. Correspondingly, the function F(x) shows no interaction of order *k* between variables $x_{i_{j}},j\in \{1,\ldots ,k\}$ if it can be expressed as the sum of *k* functions, $f_{\setminus i_{j}},j\in \{1,\ldots ,k\}$, where each $f_{\setminus i_{j}}$ does not depend on $x_{i_{j}}$:

$$F(x)=\sum_{j=1}^{k}f_{\setminus i_{j}}(x_{1},\ldots ,x_{i_{j}-1},x_{i_{j}+1},\ldots ,x_{p}).$$

In this setting, an interaction means that the effect of interacting variables is different from the sum of their marginal effects. Interactions can also be seen as a departure from a multiplicative model, in which case a log-transform enables to come back to the additive definition [13].

### 2.1. Logistic lasso-based methods

The hierNet method [6] is based on a modified version of the all-pairs lasso, that includes constraints to respect hierarchy principles: an interaction is included in the model only if one or both of its variables are marginally important. For the logistic lasso regression setting, the regular all-pairs lasso writes

(1) $$\begin{array}{rl} & \min_{{\text{β}}_{0}\in R,\text{β}\in R^{p},\Theta \in R^{p\times p}}\;q({\text{β}}_{0},\text{β},\Theta )+\lambda ||\text{β}|{|}_{1}+\frac{\lambda }{2}||\Theta |{|}_{1}\;s.t.\;\Theta =\Theta^{T},\\ & \;\mathrm{with}\;q({\text{β}}_{0},\text{β},\Theta )=-\sum_{i=1}^{n}y_{i}\log(p_{i})+(1-y_{i})\log(1-p_{i})\\ & \;\mathrm{and}\;\log\frac{p_{i}}{1-p_{i}}={\text{β}}_{0}+x_{i}^{T}\text{β}+\frac{1}{2}x_{i}^{T}\Theta x_{i}, \end{array}$$

where ${\text{β}}_{0}$ is the intercept, β is the coefficient vector for main effects, Θ the one for pairwise interactions, and λ≥0 is a penalty parameter. Note that Θ is a symmetric coefficient matrix with an empty diagonal. The authors provide the possibility to add quadratic terms $X_{j}^{2}$ by removing the empty diagonal constraint, but in our study all covariates take values 0 or 1 thus these terms are not needed. The final expression of the constraint is modified by the authors to enforce hierarchy, as well as ensuring convexity of the problem and convergence of the optimization procedure (see the original article [6] for technical details):

$$\begin{array}{rl} \underset{{\text{β}}_{0},{\text{β}}^{\pm },\Theta }{\min}\; & q({\text{β}}_{0},{\text{β}}^{+}-{\text{β}}^{-},\Theta )+\lambda 1^{T}({\text{β}}^{+}+{\text{β}}^{-})+\frac{\lambda }{2}||\Theta |{|}_{1}\\ s.t.\; & \Theta =\Theta^{T},\begin{array}{c} ||\theta_{j}|{|}_{1}\leq {\text{β}}_{j}^{+}+{\text{β}}_{j}^{-}\\ {\text{β}}_{j}^{+}\geq 0,\;{\text{β}}_{j}^{-}\geq 0 \end{array}\}\forall \;j, \end{array}$$

where $1\in R^{p}$ is a vector of ones. This formulation enables to enforce strong hierarchy, and the relaxation of the symmetry constraint on Θ allows to enforce weak hierarchy. In the strong hierarchy case, ${\hat{\theta }}_{\mathrm{jk}}={\hat{\theta }}_{\mathrm{kj}}$ by symmetry, thus an interaction will be present if and only if ${\hat{\theta }}_{\mathrm{jk}}\neq 0\;\mathrm{and}\;{\hat{\theta }}_{\mathrm{kj}}\neq 0$, meaning that both main effects ${\hat{\text{β}}}_{j}\;\mathrm{and}\;{\hat{\text{β}}}_{k}$ are present in the model. Further, a weak hierarchy interaction occurs between $X_{j}$ and $X_{k}$ if and only if ${\hat{\theta }}_{\mathrm{jk}}+{\hat{\theta }}_{\mathrm{kj}}\neq 0$, which is true if and only if ${\hat{\theta }}_{\mathrm{jk}}\neq 0\;\mathrm{or}\;{\hat{\theta }}_{\mathrm{kj}}\neq 0$, leading to ${\hat{\text{β}}}_{j}\neq 0\;\mathrm{or}\;{\hat{\text{β}}}_{k}\neq 0$ for the corresponding main effect coefficients, which means that at least one of the main effects must be in the model, but not necessarily both.

The glinternet method [19] also builds a logistic regression model including main effects and pairwise interactions, enforcing a hierarchy, but it is based on a group-lasso model rather than the all-pairs lasso. The group lasso is a generalisation of the regular lasso, where it groups the coefficients into *J* groups and applies a different penalty to each group. For logistic regression with categorical covariates, the final form of the problem is

$$\underset{{\text{β}}_{0},\text{β}}{\min}q({\text{β}}_{0},\text{β})+\lambda \sum_{j=1}^{J}\gamma_{j}||{\text{β}}_{j}|{|}_{2},$$

with $q({\text{β}}_{0},\text{β})$ on the same form as in (1), but with $\log(p_{i}/(1-p_{i}))={\text{β}}_{0}+\sum_{j=1}^{J}X_{i,j}{\text{β}}_{j}$, where *λ* is a general penalty, $X_{i,j}$ is the *i*th observation of features for interaction group *j*, ${\text{β}}_{j}$ is the corresponding coefficient vector and $\gamma_{j}$ is a penalisation constant specific to group *j*. Each group may include individual covariates or pairs of covariates representing pairwise interactions. If the coefficient vector ${\text{β}}_{j}=[{\tilde{\alpha_{1}}}^{j}\;{\tilde{\alpha_{2}}}^{j}\;\alpha_{1:2}^{j}{]}^{T}$ is non-zero, then both the main effects and the interaction are non-zero, which corresponds to the strong hierarchy principle. The whole penalty for two binary features 1 and 2 is expressed as

$$\lambda \left(||\alpha_{1}|{|}_{2}+||\alpha_{2}|{|}_{2}+\sqrt{L_{1}||{\tilde{\alpha }}_{1}|{|}_{2}^{2}+L_{2}||{\tilde{\alpha }}_{2}|{|}_{2}^{2}+||\alpha_{1:2}|{|}_{2}^{2}}\right),$$

where $L_{1}$ and $L_{2}$ are chosen to put ${\tilde{\alpha }}_{1},{\tilde{\alpha }}_{2},\alpha_{1:2}$ on the same scale. Hence, the main effects are given by $\alpha_{1}+\tilde{\alpha_{1}}$ and $\alpha_{2}+\tilde{\alpha_{2}}$, and the interaction effect by $\alpha_{1:2}$. With *p* variables, this process creates p+(p2) groups in total, with overlaps. Several runs of the method are made with a penalty parameter *λ* decreasing to gradually increase the number of covariates. This selection enables to consider several thousand covariates without scaling issues. At each penalty value, the parameters of the group-lasso are computed via the fast iterative soft thresholding approach [5].

The hierNet and the glinternet methods use a different strategy to ensure the strong hierarchy principle: while hierNet keeps the all-pairs lasso, but introduces some constraints on the coefficients, glinternet uses a group lasso where groups are made in respect of the hierarchy.

### 2.2. Logic regression-based methods

Another group of methods is based on logic regression, that was introduced by [29]. The model for Y|X is then a generalised linear model

$$g(P(Y=1\;|\;X=x))=b_{0}+\sum_{i=1}^{N}b_{i}L_{i},$$

where the $b_{i}$s are model parameters and the $L_{i}$s are Boolean combinations of the binary covariates $X_{i}$, also called logic trees. As *Y* is binary in our case, we will use the logit link function for *g*. An example of a logic tree is $L_{1}=X_{1}\cap (X_{2}\cup X_{3})$, where $X_{1},X_{2},X_{3}$ are the leaves of $L_{1}$, which expresses an interaction between $X_{1}$ and $X_{2}$ and between $X_{1}$ and $X_{3}$. The user sets the model size, i.e. the number of logic trees and the number of leaves, and simulated annealing (see, e.g. [23,39]) is then used to create logic trees with the best possible covariates. At each step one tree is selected and a small modification of this tree is picked at random following a prespecified distribution on a set of moves: alternating a leaf (changing the covariate or taking its negation), permuting operators OR and AND, growing or pruning the tree by adding new branches or selecting sub-branches, splitting or deleting leaves. For some predefined score function, for instance the binomial deviance, the acceptance probability $p_{\mathrm{acc}}$ for the new tree depends on the score improvement and the iteration time, according to

$$p_{\mathrm{acc}}=\min\left\{1,\exp([\epsilon_{\mathrm{old}}-\epsilon_{\mathrm{new}}]/T)\right\},$$

where *T* is a temperature that decreases with time, $\epsilon_{\mathrm{old}}$ is the best score so far, and $\epsilon_{\mathrm{new}}$ is the proposed new score, where smaller scores are better. During this process, model parameters are fitted at each step using the binomial deviance for logistic regression. Interactions are found by studying which variables appear together in the trees of the fitted model.

There are several versions of this method. In Monte Carlo logic regression (MCLR) [18], several runs of the method are generated to obtain a large number of models, and summary statistics of the variables present in the trees tell which are interacting most frequently. In [31], the logic feature selection procedure (logicFS) fits logic trees to several bootstrap samples of the data, and uses a measure of importance to detect interactions between co-occurring features. More recently, [16] have proposed Bayesian logic regression (BLR), which uses priors on the model size for the computation of posterior probabilities of next models. They use a genetic algorithm to generate new trees with the following moves: (i) crossover: two trees can be combined together to create a new one, (ii) mutation: one tree can be combined with a main effect that was not selected in the first step of the algorithm, and (iii) reduction: leaves can be removed from a tree to create a smaller tree. This allows to explore a larger space of solutions than with original logic regression approaches. A mode jumping MCMC procedure is used to compute the probabilities. Hyper-parameters limiting the size of models (number of leaves and trees) and the number of iterations are set by the user beforehand.

Logic regression based methods are theoretically able to explore the whole space of interactions with their search algorithms and tree-growing procedures. However, cross-validation of hyperparameters is time-consuming.

### 2.3. Neural networks and tree-based methods

[38] have proposed a neural interaction detection method (NID) based on fitting a fully connected feed-forward neural network with hidden layers to data, and then studying its weights to retrieve interactions between covariates. The method distinguishes between weights leading to the first hidden layer, and weights coming out of it. The authors assume that the former are related to interaction strength, while the latter measures the actual influence of the interaction on the model. Both types of information are then combined to give the total interaction strength in the prediction, according to the following procedure. For a network with *L* hidden layers, the number of hidden units in the *l*th layer is denoted by $p_{l}$, with $p_{0}=p$ the number of input features (covariates), and the matrix of weights coming to this layer by $W^{\left(l\right)}\in R^{p_{l}\times p_{l-1}}$. First, for a given interaction I⊂{1,…,p}, the interaction strength at a given hidden unit *i* of the first layer is calculated as $\mu (|W_{i,I}^{\left(1\right)}|)$, where μ(⋅) is an averaging function over the feature weights $W_{i,I}^{\left(1\right)}$ coming to this unit. The authors explore different options for *μ* and find that minimum over the weights is the most optimal in their settings. Then, the impact of the interaction at the hidden unit *i* of the first layer is computed using the aggregated weight of all related weights in the rest of the network

$$z_{i}=|w^{y}{|}^{T}\cdot |W^{\left(L\right)}|\cdot |W^{\left(L-1\right)}|\cdot \cdots \cdot |W_{*,i}^{\left(2\right)}|,$$

where $w^{y}$ is the vector of coefficients for the final output, all operations being made with the absolute values of the matrix entries. Finally, the total interaction strength on the outcome, at the *i*th unit of the first hidden layer is given by

$$w_{i}(I)=\mu (|W_{i,I}^{\left(1\right)}|)\cdot z_{i}.$$

Once interaction strengths are computed for every hidden unit of the first layer, the top-ranked interactions of every order, two to *p*, are selected for each hidden unit. Respective strengths by interaction are then summed across all units to obtain the final interaction strengths and rank them relative to each other, all interaction orders together. At the end we can retrieve the top *K* interactions ranked by strength, for pairwise and for any order interactions (2 separate lists).

An advantage of this method is that neural networks are in principle able to learn interactions on any form. Further, NID is gradient-based, and it is much faster than other interaction detection methods, because one does not have to search the whole interaction space. A disadvantage is that only one interaction of every order is selected by the hidden units of the first layer, forcing this layer to be large enough not to miss potential interactions.

The iterative random forest algorithm (iRF, [4]), was developed to extract interactions from a random forest [7] made of trees fitted to different bootstrap samples of the data. For each bootstrap sample, the corresponding decision tree is grown by successively splitting the tree and adding data features at the splits.

First, feature-weighted random forests RF $\left(w^{k}\right)$ are grown on the data for k=1,…,K. In step *k*, the feature-weighted random forest with weights $w^{k}=(w_{1}^{k},\ldots ,w_{p}^{k})$ is constructed by selecting feature *j* with probability proportional to $w_{j}$ at each split. For *k* = 1, the weights are uniform. For a given *k*, the mean decrease in Gini impurity of the features is computed and used as weights for step *k* + 1. Next, *B* bootstrap samples from the data are generated, and for each b=1,…,B, a random forest with weights $w^{K}$ is fitted. For each of these *B* random forests, a generalised Random Intersection Tree algorithm (RIT) is applied to extract a set of candidate interactions $S_{\left(b\right)}$, by looking for combinations of features that appear more frequently than others in a given class. Then, the stability score of each interaction *S* is computed, as the proportion of times it appears as an output of the RIT, i.e.

$$\mathrm{stability}(S)=\frac{1}{B}\sum_{b=1}^{B}\text{𝟙}\left\{S\in S_{\left(b\right)}\right\},$$

where 𝟙 denotes the indicator function.

Several other methods for detecting interactions have been proposed. Factorization based techniques used for example in recommender systems to detect user-item interaction have been considered [26], but they do exhaustive search of all pairwise combination, which makes them unfit to use with many covariates. Other techniques such as JigSaw [10] or Mean Decrease Impurity [20] could have been relevant but have no reliable code.

### 2.4. Simulation setup

To simulate immune receptor sequences, we use the OLGA software [32]. It is based on the probability distribution of V(D)J sequence recombination, a genetic mechanism by which the human body produces immune cells [37], mimicking the true distribution of cells in the body. We use command line instructions to generate the sequences, with option ‘humanIGH’ for the default human B cell heavy chain model. This generates sets of amino acid sequences corresponding to the CDR3 (complementarity-determining region 3) part of the antibodies. As the interaction detection methods require binary input of fixed dimensions, we only selected sequences of length *L* = 15, which is a frequent length in OLGA. We further performed one-hot encoding to obtain 300 binary covariates $X_{i}$, of which one, more specifically the amino acid *M*, is chosen to be the reference in order to avoid collinearity. Moreover, we removed columns where the proportion of 0s or 1s is below 0.5% to avoid convergence problems. This low value ensures that the interacting covariates are not mistakenly taken away. The final data matrix for the various cases here considered then contains around *p* = 215 covariates, and we select either 1000 or 10,000 sequences per dataset. Keeping only sequences of length *L* = 15 and then implanting interactions as described below modifies the dataset and in particular the probability distribution compared to the outcome of laboratory experiments, but this allows us to benchmark the statistical methods in a controlled setting. The modifications made are kept to the strictly necessary.

The response *Y* has been simulated according to two different models, illustrated in Appendix Figures A4 and A5: a discriminative and a generative one.

#### 2.4.1. Discriminative model

In discriminative models, after generating the covariate matrix X, we draw the response *Y* from the Bernoulli distribution with probability $p_{x}=P(Y=1\;|\;X=x)$ for each observation *x*, given by the following logistic regression model:

(2) $$F(x)=\log\left(\frac{p_{x}}{1-p_{x}}\right)={\text{β}}_{0}+\sum_{j\in S_{m}}{\text{β}}_{j}x_{j}+\sum_{i\in S_{I}}{\text{β}}_{i}\prod_{l\in i}x_{l}+\sum_{n\in S_{n}}{\text{β}}_{n}x_{n}+\epsilon ,$$

where interactions are represented as products of the binary covariates, thus enforcing non-additivity. The *β*s are model coefficients, and ${\text{β}}_{0}$ is chosen to have a class balance of 50% when the interaction is implanted in 50% of the dataset. $S_{m}$ is the set of main effects, $S_{I}$ the set of interactions, and $S_{n}=(X_{1},\ldots ,X_{p})\setminus S_{m}$ the set of weak effects covariates. Extra randomness is included in the model as weak effects $\sum_{n\in S_{n}}{\text{β}}_{n}x_{n}$, which are not essential to the binding, and a noise term ϵ∼N(0,0.01) to account for other possible effects in the binding process. The variance of the noise is set to 0.01, which is low, in order to keep the signal to noise ratio rather high.

We draw 5 main effects at random from different positions to create $S_{m}$, the rest being weak effects $S_{n}$, and interactions in $S_{I}$ are drawn from main effects or weak effects, depending on the type of hierarchy used. The main effects are chosen from positions 4 to 12 that have the most variability in the sequence. The coefficients for main effects ${\text{β}}_{j}$ are drawn uniformly from {−L,+L}, and the coefficients for interaction strength ${\text{β}}_{i}$ are set to one of {1L,2L,4L,8L}, resulting in cases where main effects will and will not override interaction effects. This happens when summing all effects together in Equation (2). For instance, with 5 main effects and an interaction strength of 8, whatever the value of main effects the sign of the sum, and thus the probability of binding, will be decided by the interactions alone. If the interaction strength is lower, both main effects and interactions will affect the probability of binding. The coefficients ${\text{β}}_{n}$ for the weak effects are drawn from the normal distribution N(0,0.01L). The value of *L* is set to have a Bayes error around 2.5% when computing the response *Y*. In the rest we will omit this value and only focus on the relative strength of interactions with respect to main effects.

All $X_{i}$s being binary, the behaviour of this model mainly depends on the value of the main effect and interaction coefficients. The contribution of a single main effect amino acid will be −1, 0, or +1 in the sum $\sum_{j\in S_{m}}{\text{β}}_{j}X_{j}$, and the total contribution of main effects will be between −5 and +5 for 5 main effects, and closer to 0 in most models. For one interaction, its contribution will be exactly ${\text{β}}_{i}\in \{1,2,4,8\}$. Hence, a clear distinction between both effects can be made when the interaction strength is higher than 5, while we may have an inconclusive assessment for lower values.

In the original OLGA data, given pairs of amino acids are not commonly present at given positions. Typically, a combination of two amino acids from randomly selected positions might be present in around 2% of the sequences, which would lead to a similar proportion of the response being non-zero with our modelling, and hence a severe class imbalance. We thus choose to implant interactions in the simulated sequences. Implantation means that we modify the type of amino acids in specific portions of the sequences to a new type. For example, if we implant the motif TT in positions 5 and 6 of the sequence CAGCAGCAGCAGCAG, it will become CAGCTTCAGCAGCAG. Note that implanting the interaction in less than 50% of sequences leads to an average proportion of binding sequences somewhat larger than the implantation rate, due to sequences that already contained the motif. However, the difference is about a few percentage points and upper bounded by the Bayes error. With this procedure for generating data, the total Bayes error remains constant ( 2.5%) for different rates of implantation (see the first section of the Appendix for more details).

The concept of hierarchy is present in several methods, in particular hierNet and glinternet. We therefore simulate different hierarchies, namely strong (all interacting amino acids are taken from main effects), weak (one or more of the interacting amino acids are taken from the main effects) or no hierarchy (interacting amino acids are taken from the weak effect covariates) to study here whether this has an impact on detection performance.

#### 2.4.2. Generative model

Generative models for data generation have been used in several studies, such as generative adversarial networks (GANs) for network traffic data generation [2], or diffusion models [41]. We present here a generative model for generation of immune data with statistical interactions. While this model is much more straightforward than GANs or diffusion models, it allows construction that does not require a large amount of immune data, and permits a direct access to the true interactions in the model in order to assert later that they are recovered correctly.

When we draw data according to the generative model, we draw X | Y=y for *y* = 0, 1. We thus create two different datasets, one with binder and one with non-binder sequences. The former is constructed so that it contains sequences with the interactions, which can be done either by implanting a given motif in binder sequences, or by rejection sampling, keeping only the sequences that contain the given motif until we obtain a desired sample size. In both cases, we also allow each dataset to contain a few sequences ( 2.5%) that would normally belong to the other dataset, so as to have a Bayes error of 2.5%, as in the discriminative case.

Note that in implantation we only change the distribution of amino acids at the position of the motif, while in rejection we change the distribution of the whole sequence when selecting one with the motif. Rejection sampling mimics naturally occurring sequences, however the technique is very computationally demanding, as the vast majority of generated sequences do not contain the interaction motif. As the results were quite similar for these two ways of generating data, we proceeded with the one based on implantation.

Similarly to the discriminative case, we can establish here the non-additivity of the function F(X) used to classify the response *Y*. Using Bayes' formula, and denoting P(Y=1)=π, the probability of classification can be written as P(Y=1 | X=x)=P(X=x | Y=1)⋅π/P(X=x) such that the log-odds ratio is given by

$$F(x)=\log\left(\frac{P(Y=1\;|\;X=x)}{1-P(Y=1\;|\;X=x)}\right)=\log\left(\frac{\pi }{1-\pi }\right)+\log\left(\frac{P(X=x\;|\;Y=1)}{P(X=x\;|\;Y=0)}\right).$$

If we have conditional independence $P(X=x\;|\;Y=y)=\prod_{i}P(X_{i}=x_{i}\;|\;Y=y)$ for *y* = 0 and *y* = 1, then the log-odds ratio can be decomposed into a sum of terms that only involve one covariate at a time, i.e. only main effects. However, when we introduce a motif, say $S_{I}=\{\{i_{1},i_{2}\}\}$, then $P(X_{i_{1}}=x_{i_{1}},X_{i_{2}}=x_{i_{2}}\;|\;Y=1)$ will not in general simplify into $P(X_{i_{1}}=x_{i_{1}}\;|\;Y=1)\cdot P(X_{i_{2}}=x_{i_{2}}\;|\;Y=1)$, and a term $F_{i_{1},i_{2}}(x_{i_{1}},x_{i_{2}})$ appears in the formula, hence the presence of an interaction.

As opposed to the discriminative case, the main effects and interactions are implicit here, and we do not know exactly what we can expect the methods to find. For instance, a motif made of three amino acids corresponds to three main effects, three two-way interactions and one three-way interaction, but their strength is unknown.

In the obtained dataset (X,Y), sequences where the interaction has been implanted will correspond to the positive class *Y* = 1, and those without the interaction to the negative class *Y* = 0. Finally, in order to study the robustness of the methods, we have explored the effect of the order of interaction (from two to four), the number of interactions (one or two), the number of sequences sampled ( $10^{3}$, $10^{4}$, $10^{5}$) and the interaction implantation rate ( 50%, 20%, 10%, and 5%).

#### 2.4.3. Software details

We use R [25] to encode our data generation procedure and run the available packages for interaction detection, and Python for the NID method. For the lasso-based methods, we use the R packages glinternet and hierNet available on CRAN. We use cross-validation to find an optimal penalisation parameter, $\lambda =10^{-4}$ for glinternet and λ=30 for hierNet, while all other parameters are kept at their default values in glinternet, and in hierNet we set diagonal=FALSE to remove pure quadratic terms $X_{i}^{2}$, and rho=nrow(x)/2 to avoid convergence issues and speed up the computation. The R packages LogicReg on CRAN and logicFS on Bioconductor.org enable to run MCLR and logicFS respectively. For both simulated annealing procedures, we set the upper and lower temperatures to 5 and 0, respectively, on a $\log_{10}$ scale, and the number of iterations to 50,000, as it is adapted to our data. For the MCLR procedure, we set nburn=10,000, niter=5,000,000, hyperpars=log(2), and ntrees=5 (the maximum possible), and in the logicFS procedure we set B=200 and ntrees=5, as suggested by the authors. Bayesian Logic Regression is run with the R package EMJMCMC available on Github (https://github.com/aliaksah/EMJMCMC2016) on 20 cores. We use the Gaussian family with Jeffrey's prior, and set probabilities p.and to 0.9, p.not to 0.05 and p.surv to 0.1, with a report level at 0.5, as suggested by the author of the method. For the NID method, (https://github.com/mtsang/neural-interaction-detection) we used the Binary Cross Entropy loss function (BCEWithLogitsLoss on PyTorch) and three hidden layers with 100, 60 and 20 neurons each, without a main effects net. We use a learning rate of $10^{-2}$ and a L1 constant of $5\cdot 10^{-4}$, and based on authors usage we divide our data into 3 sets of equal sizes for the internal training, validation and testing steps of the neural network. For the iRF method, available on CRAN, we do five iterations and compute interactions at the last one, based on authors' recommendation in their article. Since tuning of the hyperparameters is computationally demanding, cross-validation of the 2 lasso-based methods, the iterative Random Forests and the 3 logic-regression based methods has been performed on a setting with one two-way interaction present in 50% of the data, for $10^{4}$ sequences, and the penalty values and model sizes found were kept for all the other settings. We used the default number of folds of the packages *k* = 10, and *k* = 5 for the iterative Random Forest. In the discriminative setting, we used by default a strong hierarchy, with 5 main effects and an interaction strength of 8 so that the interaction effects dominate all main effects.

#### 2.4.4. Evaluation of the methods

For each dataset, we record the top 10 retrieved interactions $\hat{I_{j}^{i}},j\in \{1,\ldots ,10\}$, where *i* indexes the dataset, and *j* indexes among the detected interactions. We also record the running time and the model settings. Interactions and main effects are sorted by decreasing strength. To average out random effects, we perform 100 experiments for each setting, where the dataset and model seeds change but the model settings remain the same. We consider several performance metrics for detection ability. The most intuitive is the number of datasets $S_{\mathrm{top}1}$ (out of 100) in which the true interaction (the one that we implant) is ranked first by the method. We can also investigate in $S_{\mathrm{top}10}$ whether the true interaction is present among the top 10 retrieved. Thereafter, we can consider (for order more than two) the number of datasets $S_{\mathrm{top}1}^{\mathrm{sub}}$ in which one of the true sub-interactions is ranked first by the method. The selection of 10 interactions is a compromise between the need for flexibility in case the model is not fully optimal, and the desire to focus only on interactions that are actually important in the model. As the metrics used by each method to rank interactions differ, we could not use for instance a statistical test to set a common threshold. When logistic regression is applied, we also record the area under the receiver-operating characteristic curve (AUC) of the fitted model.

## 3. Results

We have generated synthetic immune receptor sequences, with specific interactions corresponding to implanted motifs in the chain that determine whether they are binders or non-binders, and then assessed whether the different methods can detect these interactions.

**Figure 1.**
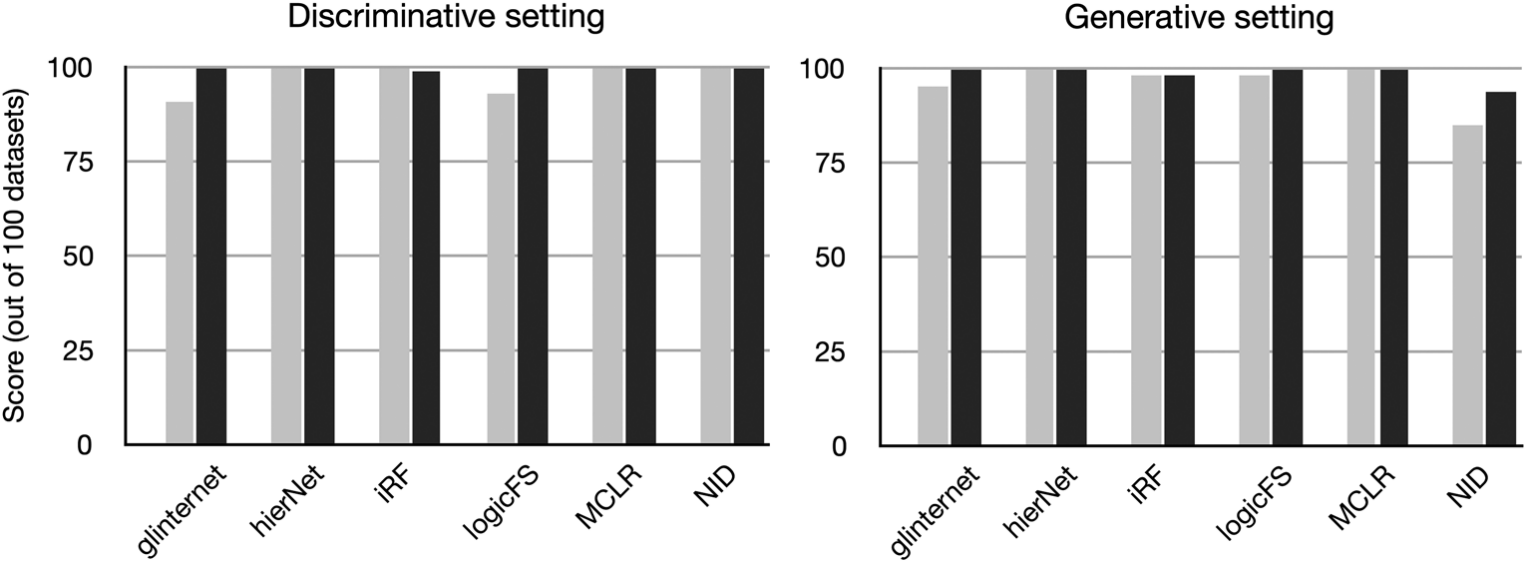
Number of datasets in which a two-way interaction is ranked first ( $S_{\mathrm{top}1}$), for 1000 (grey) and 10,000 (black) sequences. Detection is very accurate even with a low number of sequences in the dataset.

First, we simulated data with one two-way interaction present in 20% of sequences from both the discriminative and the generative models. The results, averaged over 100 datasets of size either 1000 or 10,000, are displayed in Figure 1, and show that increasing the number of sequences leads to a slightly better detection in general, but most methods detect the two-way interaction in more than 80 datasets out of 100, even with sample size only 1000. Further, the detection performance of the methods is virtually the same on data from the discriminative and the generative models, except NID, that performs slightly worse on the latter. Hence, a unique interaction pattern in the data can be detected with a few thousand sequences already, which corresponds to the typical size of available immune data. Due to great computational costs, we adopted the BLR method (not shown in the figure) on only 10 datasets and the pairwise interaction was consistently correctly retrieved for 1000 sequences, and for 8 datasets for 10,000 sequences in the sample.

**Figure 2.**
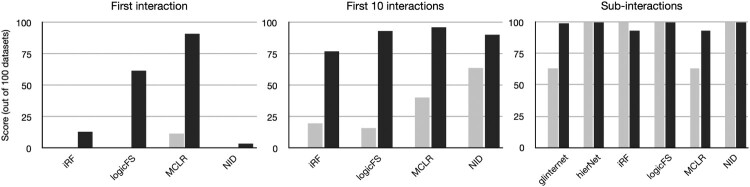
Number of datasets in which a three-way interaction is ranked first ( $S_{\mathrm{top}1}$, left), among the top 10 ( $S_{\mathrm{top}10}$, middle), or a sub-interaction is ranked first ( $S_{\mathrm{top}1}^{\mathrm{sub}}$, right), for 1 000 (grey) and 10 000 (black) sequences in the discriminative setting. Detection of a higher-order interaction is less accurate, and requires a larger dataset.

Next, we tested the detection performance on data with one three-way interaction. The corresponding results are shown in Figure 2 for the discriminative model with implantation rate 20%. Similar results are observed for the generative model (see the Figure A3 of the Appendix). Note that hierNet and glinternet are not included for the three-way interaction in the left and middle panels regarding $S_{\mathrm{top}1}$ and $S_{\mathrm{top}10}$ respectively, as they cannot detect interactions of order higher than two. We observe that the detection performance is overall much worse than for the simpler two-way interaction. When using 1000 sequences, only MCLR can detect the three-way interaction in the 100 datasets. However, increasing the sample size to 10,000 improves the performance of all methods. Further, if the correct interaction is not ranked first, it is still detected in the top 10 of all methods.

We also assessed whether the three sub-interactions of order two are detected. The corresponding results are shown in the right panel of Figure 2, and indicate that all methods manage to detect sub-interactions, even though they do not find the three-way one. Again, we observe a slight increase in performance when using 10,000 sequences per dataset with respect to 1000 sequences, even though hierNet, iRF, logicFS and NID provide reliable results with the smaller dataset. Most methods might not include valid interactions of greater complexity if the current model already shows good performance, thus hindering the capacity to detect high-order interactions in general. Once more, BLR was only tested on 10 datasets. The three-way interaction was retrieved correctly in one dataset for 1000 sequences, and five datasets for 10,000 sequences in the sample, and the sub-interactions in all 10 datasets and nine datasets, respectively.

The results show that high-order interactions can be retrieved, although often failing to recognise its relative importance in respect to lower-order interactions. The tree-based methods have better performance than the neural network for high-order interactions, ranking the correct interaction as first more accurately. The study of four-way interactions provided similar patterns as for the three-way interaction and are not shown here. Further, we have studied the detection when several interactions are present in the dataset at the same time, such as when one fourth of the sequences has one interaction implanted and one fourth has another interaction (results not shown). Also for this case, the performance was similar: pairwise interactions are retrieved in most datasets, while the detection ability decreases for higher-order interactions.

**Figure 3.**
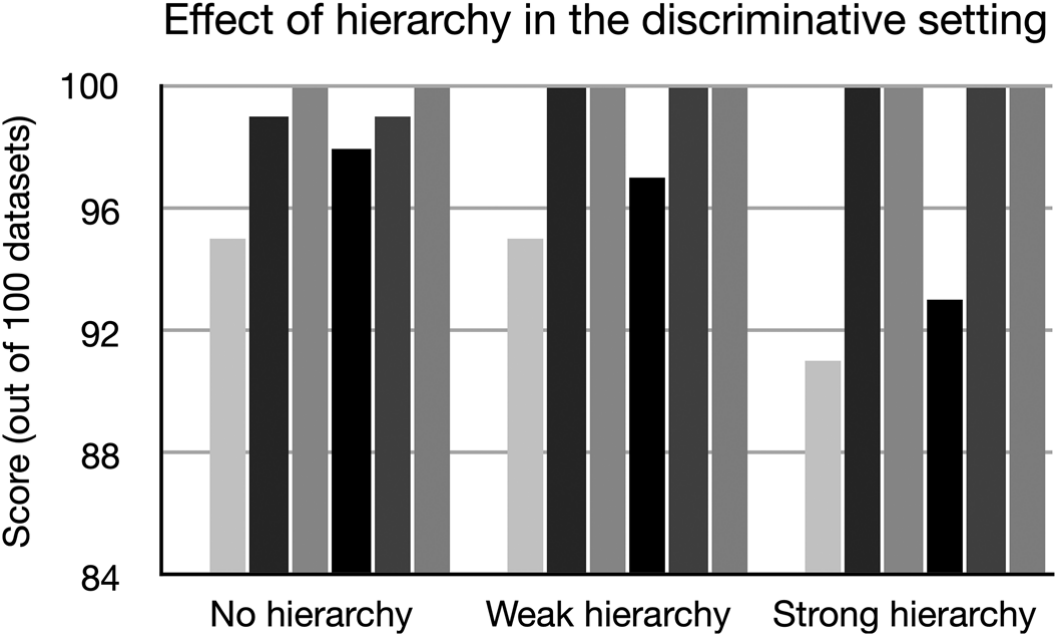
Number of datasets in which the interaction is ranked first for different hierarchies. Methods from left to right bar: glinternet, hierNet, iRF, logicFS, MCLR, NID. Performance is slightly lower in the strong hierarchy setting.

In order to study how the type of hierarchy affects the detection methods' ability to find interactions, we simulated from a discriminative model with implantation rate 20% for the 3 types of hierarchy: strong, weak, and no hierarchy. The results are given in Figure 3 for 1000 sequences. These show that when there is no hierarchy, the methods perform slightly better, even the ones assuming a hierarchy. The reason may be that when there is a hierarchy, interaction effects may to a larger extent be approximated by main effects, making them more difficult to detect.

In the discriminative setting, the influence of interaction strength (value 1, 2, 4 or 8) is explored in Table 1 for the glinternet method. The table shows the number of data sets among 100 of size 1000 for which a single implanted two-way interaction was retrieved, for different combinations of interaction strength and implantation rate. As expected, a higher strength leads to better separation from main effects, thus better detection. For a low strength, detecting the correct interaction happens less frequently, and depends on how the main effect coefficients are distributed in the model. The pattern is similar for all the other methods. Higher-order interactions are still distinguishable from main effects when the interaction strength is high, but the methods typically detect lower-order sub-interactions with the correct covariates instead.

**Table 1.** Effect of interaction strength and implantation rate on detection ability.

Implantation rate	Interaction strength			
	1	2	4	8
0.5	16	30	77	78
0.2	12	32	78	91
0.1	8	29	80	97
0.05	6	16	73	93

Note: Number of datasets out of 100 in which the glinternet method ranks the correct interaction first, for the discriminative setting.

**Figure 4.**
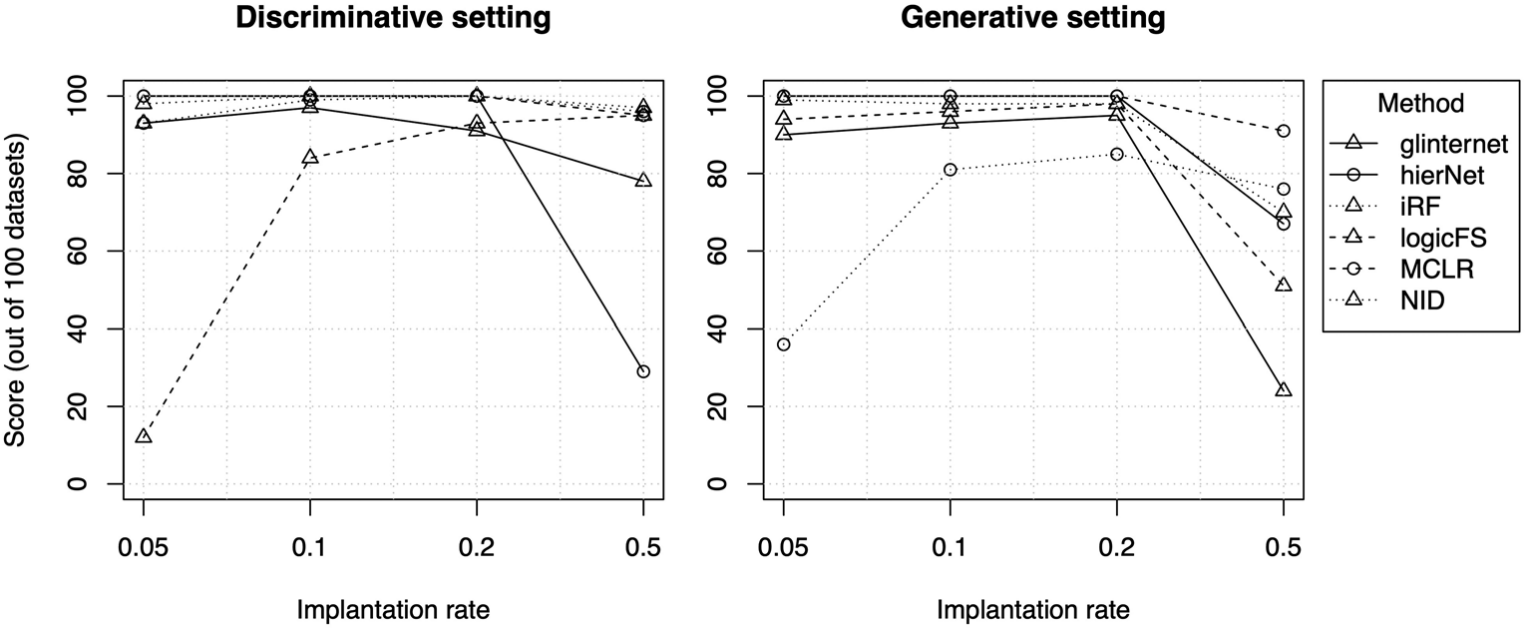
Number of datasets in which the interaction is ranked first according to implantation rate. The best performance happens when the interaction is implanted in 10% or 20% of sequences.

**Figure A2.**
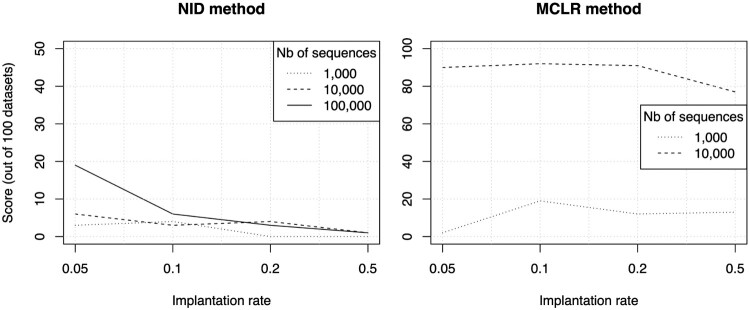
Number of datasets for which the interaction is ranked first by the NID (left) and the MCLR (right) methods, in a discriminative model with a 3-way interaction.

**Figure A3.**
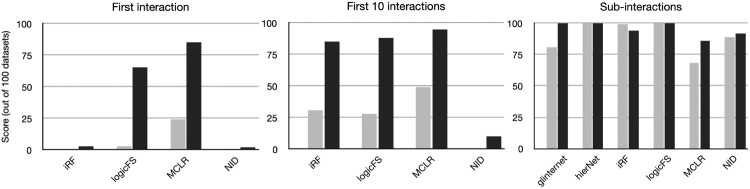
$S_{\mathrm{top}1}$, $S_{\mathrm{top}10}$ and $S_{\mathrm{top}1}^{\mathrm{sub}}$ for 1000 (in grey) and 10,000 (in black) sequences, 3-way interaction.

**Figure A4.**
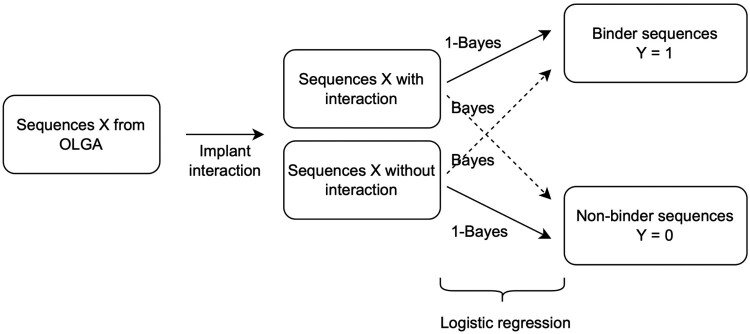
Data generating process in the discriminative model, where ‘Bayes’ denotes the Bayes error.

**Figure A5.**
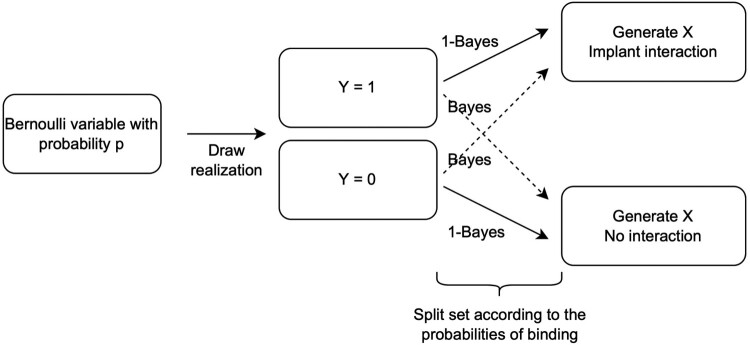
Data generating process in the generative model, where ‘Bayes’ denotes the Bayes error.

The effect of the implantation rate is generally that a higher value enables better detection, but the best detection does not always happen at the highest value. This is illustrated in Figure 4. We observe that the best performance is not for the highest implantation rate ( 50%), but rather for lower values ( 20% or 10%). For the lowest studied implantation rate ( 5%), performance decreases again slightly. This effect was also observed for a higher number of sequences and for higher-order interactions (see Appendix Figure A2 for the performance of NID and MCLR).

To understand why this happens, we fitted a logistic regression model with the true main effects, both with and without the true interaction (the one that we implant), and computed the AUC value of the fitted model on a test set in both cases. Training and test sets of equal sizes were simulated from a discriminative model, with 5 main effects and an interaction strength of 8, for different implantation rates and different types of hierarchy. The results for a two-way interaction are shown in Table 2, and are averaged over 100 different datasets. The results for a three-way interaction were very similar (not shown). The AUC values for the models with interaction effects are very close to 1 for all considered hierarchies. However, the AUC gain from including interactions differs for the various hierarchies, showing larger values in the presence of no hierarchy and smaller for strong hierarchies. This might explain why some detection methods performed better when there is no hierarchy. Further, in the setting with strong hierarchy, the gain in AUC from including the interaction is very small for the highest implantation rate of 50%, which could explain the poorer detection performance, compared to implantation rate 20%, observed in Figure 4. We also computed the AUC values using 10-fold cross-validation, for the top line of the table with 5000 sequences, and found numerical values that were very close to the ones using a training and a test set. Including cross-validation results is done for example in [33], and is useful to anticipate the generalisation power of the method on new independent datasets.

**Table 2.** Effect of hierarchy on performance of a logistic regression model with and without interactions.

		No hierarchy			Weak hierarchy			Strong hierarchy		
		Number of sequences								
Implantation rate		500	5000	50,000	500	5000	50,000	500	5000	50,000
0.5	AUC	0.98	0.98	0.98	0.98	0.98	0.98	0.98	0.98	0.98
0.2		0.95	0.96	0.96	0.95	0.96	0.96	0.95	0.96	0.96
0.1		0.87	0.93	0.92	0.88	0.93	0.92	0.88	0.93	0.92
0.05		0.75	0.87	0.87	0.75	0.87	0.87	0.75	0.87	0.87
0.5	AUC gain	0.44	0.43	0.43	2.6e−2	2.7e−2	2.7e−2	4.4e−4	8.7e−4	8.3e−4
0.2		0.41	0.41	0.41	2.6e−2	2.9e−2	2.8e−2	3.1e−3	3.4e−3	3.2e−3
0.1		0.34	0.38	0.38	2.4e−2	3.0e−2	2.8e−2	4.7e−3	7.0e−3	6.5e−3
0.05		0.22	0.32	0.32	2.9e−2	3.0e−2	2.7e−2	1.7e−2	1.2e−2	1.1e−2

Note: AUC values for the models with true main effects and interactions (top 4 rows), and AUC gain with respect to the models with only the true main effects (bottom 4 rows). The number of sequences indicates the size of the training set on which logistic regression is fit. Here, we provide the true interaction covariates so that the model does not have to search for them.

**Figure 5.**
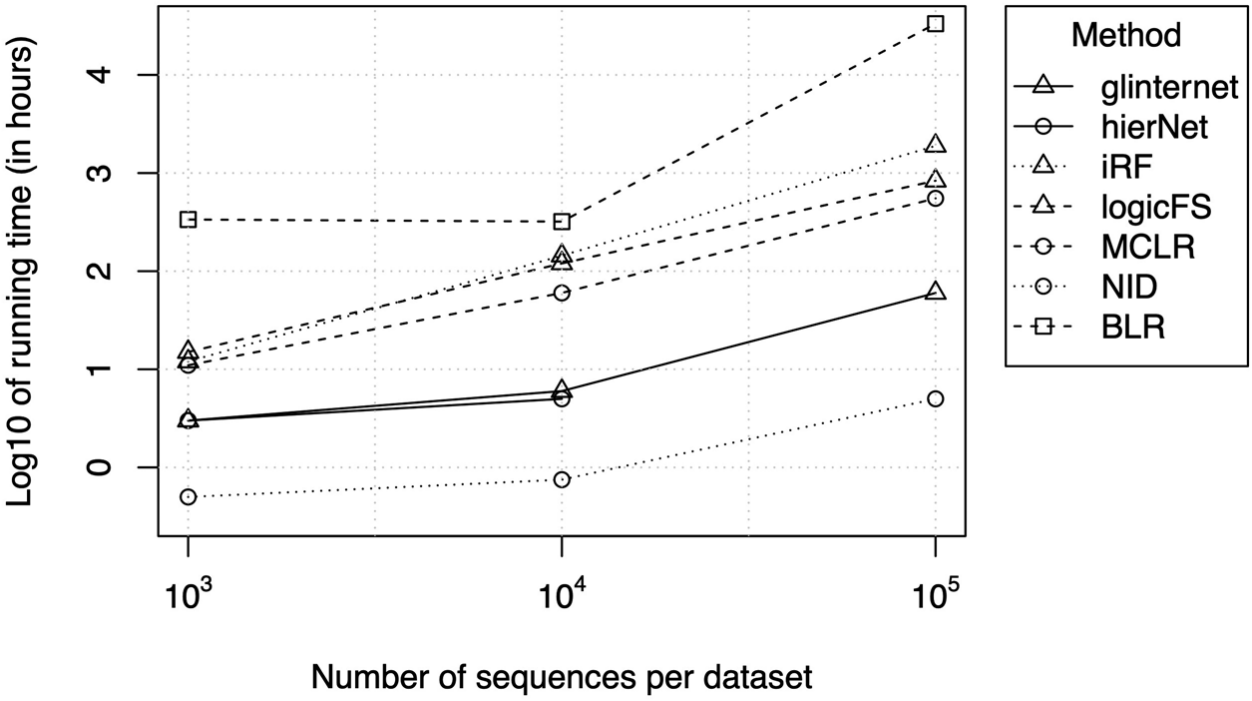
Running times for 100 datasets, computed in the generative setting.

The running time for the different methods on 100 datasets as a function of the size of the data sets is shown in Figure 5. As expected, it increases with the number of observations, except for the BLR method. Most notably, the neural network based technique (NID) is one order of magnitude faster than most other methods while showing similar detection performance. The hierNet method could not handle more than 40,000 sequences on our cluster for its exceeding memory requirements. Further, due to the computational demands, the BLR performances have been asserted upon one dataset only, resulting in a rather uncertain assessment.

### 3.1. Illustration on experimental data

We also illustrate the methods' ability to detect statistical interactions from experimental immune receptor data obtained from a study on the antigen HER2 [21]. This dataset consists of 45,361 amino acid sequences of length 15, of which 14,517 are classified as binders to HER2, and 30,844 as non-binders. The data were one-hot encoded, and we removed columns where the proportion of 0s or 1s falls below 0.01%, leaving 211 covariates. We split the data randomly into a training set with 70% of the sequences and a test set with the remaining 30%. After a cross-validation step, we ran the interaction detection methods on the training set and recorded the top 10 detected interactions for each method.

These are shown in Table 3. Interestingly, 14 pairwise interactions were found independently in the top 10 of several methods, namely 5G:6G, 5G:11A, 5G:13D, 8G:9P, 8G:11A, 8G:11K, 8G:11P, 8G:12F, 8G:12I, 9P:11A, 11A:12I, 11K:12F, 11V:12F and 12F:13D. This suggests important interaction effects between amino acids at these positions. In particular, amino acid G at position 8 seems to be required in the binding process. This is in accordance with [21], who find that 8G is almost twice as much present in binding sequences than in non-binding ones. One interaction of higher order, namely 8G:11A:12F, was found by iRF, and had all its corresponding pairwise sub-interactions also detected by at least one other method.

**Table 3.** Output of interaction detection methods on experimental data.

BLR	glinternet	hierNet	iRF	logicFS	MCLR	NID
4F:12N	8G:11K	4W:13D	8G:9F:11A:12F	!4F:8G	5G:6G	8G:11A
8A:11P	12F:13D	8G:9P	8G:10Y:11A:12F	8G:!12I	5G:13D	8G:12F
5G:11A	5G:6G	11V:12F	8G:11A:12F	!4F:!10F	6G:13D	11A:12F
6A:12I	8G:11P	12F:13D	8G:11A:12F:13D	!4F:!10F:!12I	8G:11A	8G:11K
	11V:12F	4W:13G	8G:12F	!11K:12F	8G:11P	11K:12F
∅	10F:12I	4W:5G	4F:8G:11A:12F	!4F:10Y	11P:12I	8G:12I
	11A:13D	5G:11A	4W:8G:11A:12F	11A:!12I	11V:12F	11A:12I
	10Y:11K	11P:13D	5G:8G:9F:11A:12F	!4F:8G:!12I	8G:9P	11K:12I
	5G:13D	6N:8S	5G:8G:11A:12F	8G:!11K:!12I	9P:11A	9F:11K
	8G:12I	4W:8G	5G:6G:8G:11A:12F	!9P:11A	9P:11P	8G:9F

Note: Top 10 retrieved interactions from the training set of the experimental data. In the logicFS method, an exclamation mark denotes the negation of a binary variable. The BLR method only identifies 4 interactions here.

We then fitted a logistic regression model to the training set, first with only the main effects, and then also with the detected interactions from all methods, and computed the corresponding AUC value and accuracy on the test set. We reported an AUC of 0.88 and average precision (area under the Precision-Recall curve) of 0.77 for the first model, and an AUC of 0.89 and average precision of 0.79 for the second. Hence, the improvement of the model from including interactions was only minor in this case, but they might still provide insight into the binding process. Further, it is more robust than fitting all candidate pairwise interactions in a logistic regression model: this would lead to a much longer running time and less certainty in the estimated coefficients values. In comparison, [21] had obtained an AUC of 0.91 and average precision of 0.83 on a test set with a Convolutional Neural Network. In addition to AUC values on the test set, we also computed the performance of the model using 10-fold cross-validation. The numerical values found were identical to the previous values at a precision of 2 digits.

## 4. Conclusion

Statistical interactions are present in various data generating processes. In immune receptor data, deconstructing the relative importance of main effects and interactions will help understanding the binding process of antibodies to antigens. In this paper, we have compared the performance of different interaction detection methods based on logistic lasso, logic regression, random forests and neural networks, in the immune receptor binding setting. The comparison was done on simulated immune receptor data, obtained from either a discriminative or a generative model, with an illustration on antibody data. More specifically, we tested how different factors, such as the strength of interactions with respect to main effects, the implantation rate, the order of the interactions, the sample size and the type of hierarchy affected the detection performance of the methods.

Notably, the interaction strength, together with the implantation rate, are important. Interactions must have similar or greater impact than main effects in the model and also be present in a sufficient fraction of the sequences for the detection methods to succeed. But most important is the interaction order: while pairwise interactions are retrieved in the vast majority of datasets, interactions of higher order are often confused with their sub-interactions. On the other hand, the hierarchy does not really affect the results, not even for the methods that assume certain hierarchies, and neither does the discriminative or generative setting. The sample size for detecting interactions starts with a few thousand sequences and is thus compatible with currently available sequencing data.

Pairwise interactions with a strong effect on the response were detected by all methods in most cases. For detection of higher-order interactions, only 4 methods could be used directly, namely iRF, logicFS, MCLR and NID, and the MCLR method showed the best performance at that task. When applying the methods to real-world datasets, it is important to be able to handle the large datasets that may be the target of analysis. Here, only glinternet and NID were able to handle the largest datasets of 100,000 sequences in a reasonable amount of time (less than 100 h). Altogether, NID was by far the fastest method for all sizes of datasets. The good performance of other methods such as MCLR for different orders of interactions or hierNet for pairwise interactions is mitigated by their long running time (about 60 hours for 10,000 sequences with MCLR) and inability to handle large datasets (over 500 h for 100,000 sequences for MCLR, while hierNet does not handle that size). The neural network based method (NID) has shown optimal performance at detecting pairwise interactions, and is also able to detect higher-order interactions, with a lower running time than the other methods and the ability to handle the largest datasets without scaling issues. Hence, neural-network based models are potential avenues for development and improvement of interaction detection methods. The lasso-based methods are efficient, but they are so far limited to pairwise interactions. For higher-order interactions, the tree-based methods (either using logic regression or random forests) are interesting alternatives to NID, but they have a longer running time.

We can thus successfully detect statistical interactions in immune receptor data, supporting the understanding of the receptor regions of relevance in a binding process. In experimental data, statistical interactions are less obvious but common patterns emerge when crossing the results of different interaction detection methods, and prior detection enables to improve models in a robust way. These findings are not limited to immune receptors and can be applied to other biological binding processes as well. For this, there needs to be exhaustive information on the structure composition in both cases of binding and non-binding, and enough data–at least in the thousands structures in both classes for a sequence length the same as in our study–to train the detection methods.

Immune receptor data is modelled as categorical data, which all interaction detection methods do not handle. This limits the candidate methods for analysis. However, the CDR3 part of the immune receptor plays a major role in the binding [9,12,40], which allows us to focus on a reduced portion of the receptor. In other biological systems, there might be more uncertainty on which part of the system to study for interactions, resulting in more covariates to take into account and the need for pre-selection. This is the case in genetic studies of cancer susceptibilities for example, where thousands of genes are potential candidates for susceptibility [24].

## Figures and Tables

**Figure A1.**
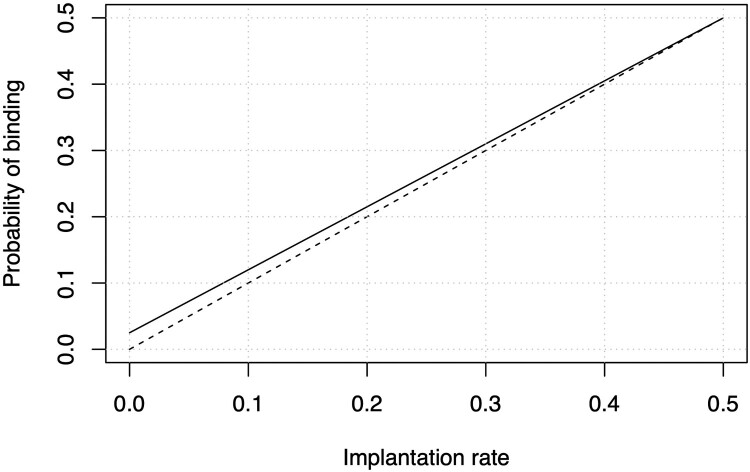
Probability of binding for different implantation rates, for a Bayes error of 2.5% (the dashed curve is the identity function).

## Appendix 1. Controlling the Bayes error in the model

A Bayes classifier is one that minimizes the probability of misclassification, and the minimal value of this probability is called the Bayes error $e_{\mathrm{Bayes}}$. In the discriminative simulation model, the response variable *Y* follows a Bernoulli distribution with parameter

$$p_{x}=P(Y=1\;|\;X=x)={\left(1+\exp(-\alpha (\tilde{{\text{β}}_{0}}+g(x))-\epsilon )\right)}^{-1}.$$

We choose the parameters α>0 and $\tilde{{\text{β}}_{0}}$ so that sequences with the interaction have a high probability of binding ( $p_{x}>0.5$), whereas sequences without the interaction have a low probability ( $p_{x}<0.5$). Since *Y* is stochastic, a few sequences with the interaction and high probability of binding will still result in *Y* = 0, and a few sequences without the interaction will result in *Y* = 1. The probability of the former case is P(Y=0 | X,E),where *E* is the event ‘ X has the interaction’, and $P(Y=1\;|\;\bar{E})$ in the latter case, where $\bar{E}$ is the complement of *E*. The corresponding misclassification errors are

$$P(Y=0\;|\;E)=E_{X}(1-P(Y=1\;|\;X)\;|\;E)=E_{X}\left({\left(1+\exp(+\alpha (\tilde{{\text{β}}_{0}}+g(X))+\epsilon )\right)}^{-1}|E\right)$$

and

$$P(Y=1\;|\;\bar{E})=E_{X}(P(Y=1\;|\;X)\;|\;\bar{E})=E_{X}\left({\left(1+\exp(-\alpha (\tilde{{\text{β}}_{0}}+g(X))-\epsilon )\right)}^{-1}|\bar{E}\right).$$

Hence, the total Bayes error when half of the sequences contain the interaction is given by

$$e_{\mathrm{Bayes}}\left(0.5\right)=\frac{1}{2}E_{X}(1-p_{X}\;|\;E)+\frac{1}{2}E_{X}(p_{X}\;|\;\bar{E})=E_{X}((1+\exp\;|\;\alpha (\tilde{{\text{β}}_{0}}+g(X))+\epsilon {)}^{-1}).$$

The last equality assumes that the distribution of sequences *X* is symmetric around 0.5, so that $E_{X}(1-p_{X}\;|\;E)=E_{X}(p_{X}\;|\;\bar{E})=e_{\mathrm{Bayes}}(1/2)$. In the simulation study, the distribution of probabilities is not exactly symmetric, but not too far from this ideal case.

We tune the parameters so that $e_{\mathrm{Bayes}}(s=0.5)=0.025$, which corresponds to having 2.5% of sequences associated with a response contrary to whether they have the interaction or not. The class balance ensures that these sequences are divided equally between binders and non-binders. When reducing the implantation rate, the Bayes error in the data remains the same:

$$e_{\mathrm{Bayes}}(s)=sE_{X}(1-p_{X}\;|\;E)+(1-s)E_{X}(p_{X}\;|\;\bar{E})=e_{\mathrm{Bayes}}(0.5)\cdot [s+(1-s)]=e_{\mathrm{Bayes}}(0.5).$$

However, for lower implanting rates the marginal probability of binding is a little higher than the implantation rate, so that P(Y=1)>s for s<0.5, but the difference remains smaller than the Bayes error, as shown in the equation below. In Figure A1, we show the probability of binding for different implantation rates. We study implantation rates up to 5%, for which the actual probability of binding is 7.25% in this ideal model.

$$\begin{array}{rl} P(Y=1)=P(Y=1\;|\;E)\cdot s+P(Y=1\;|\;\bar{E})\cdot (1-s) & =(1-e_{\mathrm{Bayes}})\cdot s+e_{\mathrm{Bayes}}\cdot (1-s)\\ & =s+e_{\mathrm{Bayes}}\cdot [1-2s]. \end{array}$$
